# Clinical history of HIV infection may be misleading in cytopathology

**DOI:** 10.4103/1742-6413.64375

**Published:** 2010-06-12

**Authors:** Liron Pantanowitz*, Michael Kuperman, Robert A. Goulart

**Affiliations:** Address: Department of Pathology, Baystate Medical Center, Tufts University School of Medicine, Springfield, MA, USA

**Keywords:** AIDS, cytology, HIV, misleading

## Abstract

Human immunodeficiency virus (HIV)-infected patients are at an increased risk for developing opportunistic infections, reactive conditions and neoplasms. As a result, a broad range of conditions are frequently included in the differential diagnosis of HIV-related lesions. The clinical history of HIV infection may, however, be misleading in some cases. Illustrative cases are presented in which knowledge of a patient's HIV status proved to be misleading and increased the degree of complexity of the cytologic evaluation. Case 1 involved the fine needle aspiration (FNA) of a painful 3 cm unilateral neck mass in a 38-year-old female with generalized lymphadenopathy. Her aspirate revealed a spindle cell proliferation devoid of mycobacteria that was immunoreactive for S-100 and macrophage markers (KP-1, PGM1). Multiple noncontributory repeat procedures were performed until a final excision revealed a schwannoma. Case 2 was a CT-guided FNA of a positron emission tomography positive lung mass in a 53-year-old man. The acellular aspirate in this case contained structures resembling fungal spore forms that were negative for mucicarmine and GMS stains, as well as cryptococcal antigen immunocytochemistry. A Von Kossa stain confirmed that these pseudo-fungal structures were calcified debris. Follow up revealed multiple calcified lung and hilar node based granulomata. Case 3 involved the cytologic evaluation of pleural fluid from a 47-year-old man with Kaposi sarcoma and recurrent chylous pleural effusions. Large atypical cells identified in his effusion were concerning for primary effusion lymphoma. Subsequent pleural biopsy revealed extramedullary hematopoiesis, documenting these atypical cells as megakaryocytes. These cases demonstrate that knowledge of a patient's HIV status can be misleading in the evaluation of cytology specimens, with potential for misdiagnosis and/or multiple procedures. To avoid this pitfall in the setting of HIV infection, common entities unrelated to HIV infection and artifacts should always be included in the differential diagnosis.

## INTRODUCTION

Human immunodeficiency virus (HIV) causes acquired immunodeficiency syndrome (AIDS). As CD4 T-cell numbers decline, HIV-infected patients become progressively more susceptible to opportunistic infections (bacterial, fungal, viral and parasitic) and the development of malignancies. AIDS-defining cancers include Kaposi sarcoma (KS), cervical cancer and high-grade non-Hodgkin lymphoma. Patients infected with HIV may also have unusual manifestations of common infections and neoplasms, such as spindle cell tumors associated with mycobacteria. As patients infected with HIV are living longer due to the benefits of antiretroviral therapy, they are now also more likely to develop certain non-AIDS-defining cancers such as lung cancer, hepatocellular carcinoma and Hodgkin lymphoma.[1] HIV-infected patients are also at increased risk for developing specific reactive conditions, such as generalized lymphadenopathy with follicular lymphoid hyperplasia and Castleman disease.[2] As a result, a broad range of benign (reactive and infectious) and neoplastic conditions are frequently included in the differential diagnosis of HIV-related lesions. However, this wide differential might at times mislead the cytopathologist. Moreover, as conditions unrelated to HIV infection may also arise in this setting, these entities also need to be considered in the differential diagnosis of a lesion arising in the HIV positive individual. Illustrative cases are presented in which knowledge of a patient's HIV status proved to be misleading and increased the degree of complexity of the cytologic evaluation.

## CASE REPORTS

### CASE 1

**Figure 1:**
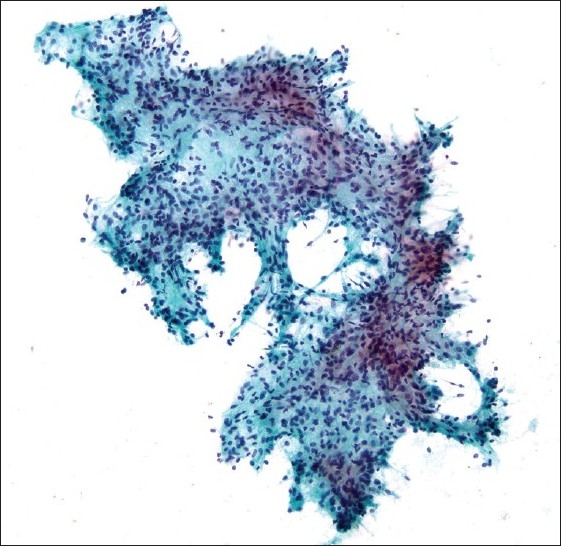
Fragment of morphologically bland spindle cells seen on direct smear (Pap stain; original magnification ×200).

**Figure 2:**
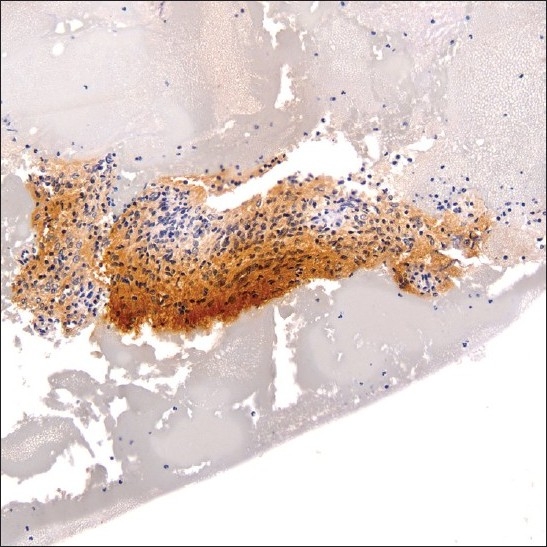
S-100 immunoreactive spindle cell fragment within cell block material (original magnification ×200).

**Figure 3:**
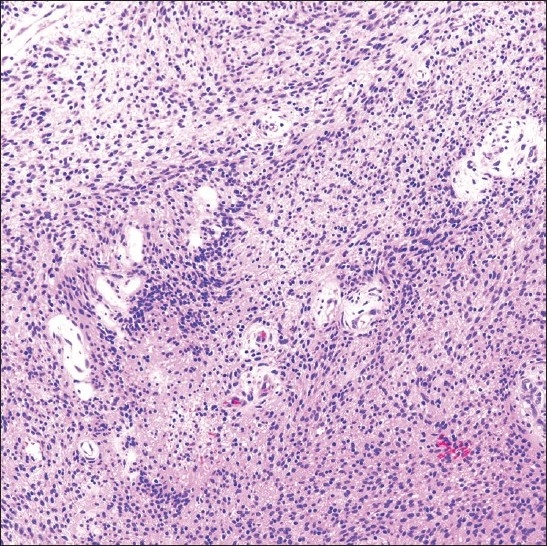
Schwannoma excision specimen showing an Antoni type A cellular area of spindle-shaped cells (H and E stain; original magnification ×200).

A 38-year-old HIV positive female with a CD4 cell count of 260 cells/mm^3^ and HIV viral load of 191,528 copies/mL presented with left neck pain. She was naïve to antiretroviral therapy. An ultrasound of her neck revealed four enlarged, hypoechoic, enhancing masses in the upper lateral neck interpreted to be lymph nodes. A fine needle aspiration (FNA) was performed that yielded scant material showing a morphologically bland spindle cell lesion [Figure 1], thought to represent a granulomatous reaction. Special stains for mycobacteria (modified Kinyon stain) and fungi (GMS stain) were negative. The patient underwent a second FNA one week later followed by an ultrasound-guided core needle biopsy. Both the FNA and core biopsy again demonstrated a spindle cell proliferation with positive immunoreactivity for S100 [Figure 2] and the macrophage markers KP1 and PGM1. Markers for epithelial membrane antigen (EMA), CD34, desmin, smooth muscle actin and keratin cocktail were negative. Repeat Kinyon and GMS stains were negative. At this stage, definitive excision was recommended. Accordingly, the patient underwent a left neck lymph node biopsy. However, while this biopsy revealed a reactive lymph node with follicular lymphoid hyperplasia, no spindle cell lesion was identified. As a result, two weeks later a neck surgical exploration was performed with removal of a retromandibular mass. Histological examination of this mass proved it to indeed be a schwannoma [Figure 3].

### CASE 2

**Figure 4:**
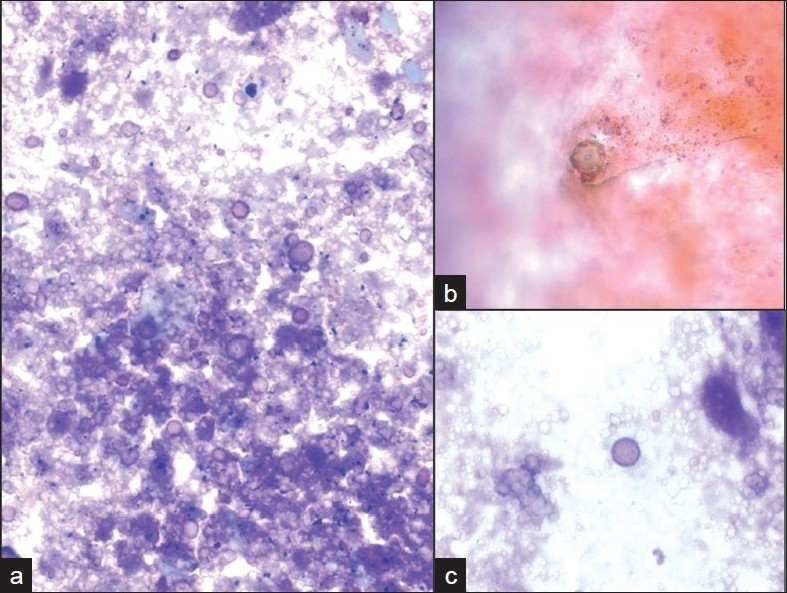
Ovoid pseudo-fungal structures among a) acellular debris with b) some that appear to have a cell wall and c) others that appear unencapsulated. (Pap stain; original magnification ×600).

**Figure 5:**
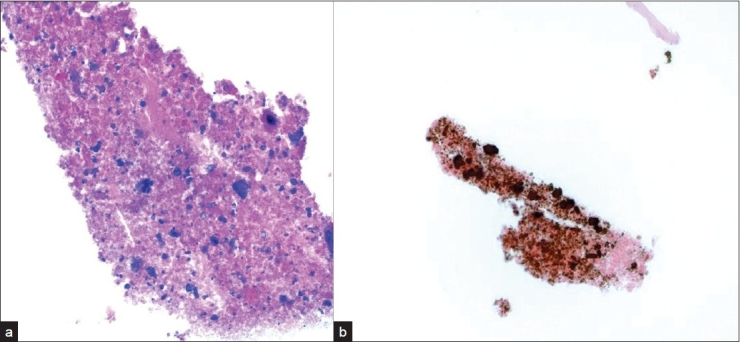
a) Acellular cell block material with fragmented calcified debris that b) stains positively with a von Kossa stain for calcium (original magnification ×200).

Incidental nodules were found on a chest X-ray in a 53-year-old HIV positive male. A follow-up positron emission tomography (PET) and CT scan raised the possibility of a neoplastic process. A lung FNA was performed one month later, which yielded an acellular specimen that was comprised of granular debris with several ovoid bodies that resembled budding fungal spore forms measuring up to 15 μm or larger [Figure 4]. Mucicarmine and GMS stains were negative. Immunocytochemistry for cryptococcal antigen was also negative. A von Kossa stain performed on cell block material was positive in calcific debris, including pseudo-fungal forms [Figure 5]. Further imaging studies revealed that the patient had multiple calcified granulomata in both lungs and within hilar lymph nodes.

### CASE 3

**Figure 6:**
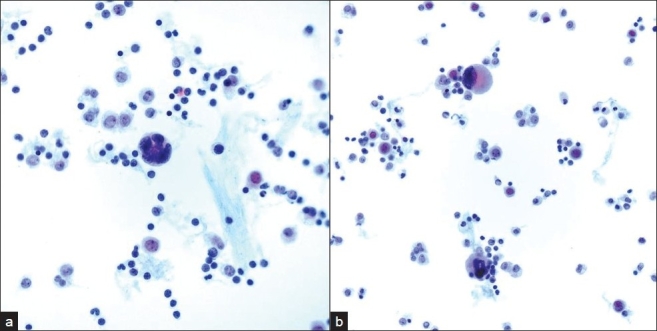
Single large atypical cells with a) hyperchromatic and b) multinucleated nuclei are shown in pleural fluid admixed with chronic inflammatory cells. (Pap stain; original magnification ×600)

**Figure 7:**
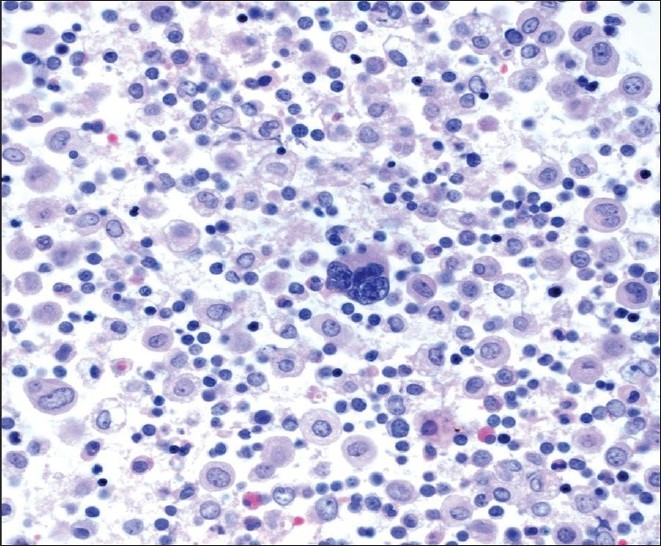
Cell block material containing a large central megakaryocyte (H and E stain; original magnification ×600).

**Figure 8:**
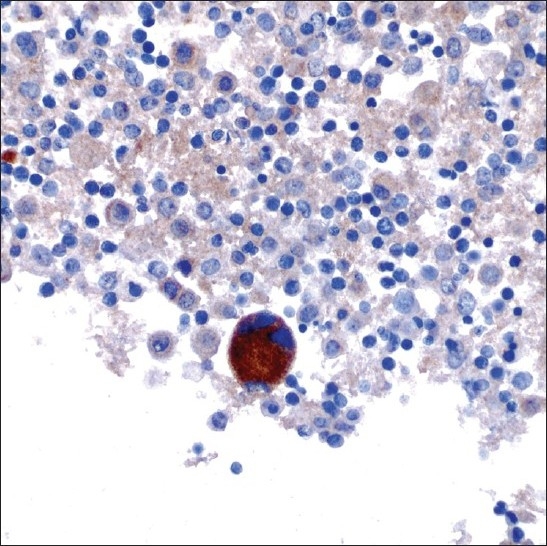
A megakaryocyte showing CD61 immunoreactivity (original magnification ×600).

A 47-year-old male patient with a past medical history significant for smoking, AIDS, anemia (hematocrit 35.3%) and KS presented with recurrent chylous pleural effusions. Multiple pleurocentesis procedures were performed with fluid submitted for cytological evaluation. The majority of cells in these effusions were reactive mesothelial cells and admixed granulocytes. Also identified in these pleural effusions [Figures 6 and 7] were scattered large cells with atypical, hyperchromatic and occasionally multinucleated nuclei. The possibility of primary effusion lymphoma (PEL) was entertained. Immunostains were performed that showed these large cells to be positive for the megakaryocytic markers factor VIII and CD61 [Figure 8], but negative for CD20, CD3 and LNA-1. LNA-1 is a biomarker for human herpesvirus-8 (HHV8) infection. A keratin cocktail stained only reactive mesothelial cells and a CD68 immunostain highlighted numerous macrophages. CD20 and CD3 stains revealed a mixed background of lymphocytes that demonstrated polytypic immunoreactivity with kappa and lambda markers. A myeloperoxidase immunostain showed frequent early myeloid (myelocytes and promyelocytes) and eosinophilic elements. Material was not submitted for flow cytometry. Modified Kinyon and GMS stains were negative for mycobacteria and fungi, respectively. A parietal pleural biopsy showed dense fibrous plaques and adhesions with extramedullary hematopoiesis, confirming that the large atypical cells were megakaryocytes. This patient was not known to have an underlying hemolytic anemia or hemoglobinopathy. He subsequently developed multiple myeloma two years later.

## DISCUSSION

Lack of pertinent clinical information provided with specimen samples that are submitted to the clinical and anatomic laboratories is a frequent complaint of pathologists. Inadequate knowledge of a patient's clinical history may hinder the pathologic interpretation. The three cases presented demonstrate how the knowledge of a patient's HIV status, in contrast, can occasionally be misleading in the evaluation of cytology specimens, with potential for misdiagnosis and/or multiple potentially unnecessary procedures. Taking into consideration the high prevalence of HIV and AIDS around the world, and limited resources for diagnosis in developing countries, cytology (e.g., FNA) has proven to be a useful method for diagnosis, reducing the necessity for surgical excision, and facilitating rapid triage for therapy.[3]

In case 1, the spindle cell lesion originally identified in the FNA material was subsequently histologically proven to represent a schwannoma. The differential diagnosis of a spindle cell proliferation/lesion in an HIV infected patient includes entities somewhat unique to HIV such as KS, mycobacterial spindle cell pseudotumor and Epstein Barr Virus (EBV)-associated smooth muscle tumors, as well as spindle cell lesions independent of HIV infection (e.g., nodular fasciitis, thymoma, mesenchymal sarcomas, etc.). Most FNA specimens of KS have a bloody background in which intact to loosely cohesive clusters of bland spindle-shaped cells are distributed.[4‐6] Closely packed KS spindle cells are usually overlapping and have indistinct cytoplasmic borders. Vascular spaces containing blood and metachromatic globular structures that correspond to the eosinophilic globules seen in histologic sections may rarely be present. In difficult cases, detection of HHV8 using the LNA-1 immunocytochemical stain may be necessary. Mycobacterial pseudotumor is an exuberant spindle cell lesion induced, mainly within lymph nodes, by atypical mycobacteria.[7‐8] As a result, acid-fast bacilli should be present in these pseudotumors. The spindle cells in mycobacterial pseudotumor are also positive for S-100 protein and CD68 immunostains. S-100 protein is not strictly specific to nervous tissue and its tumors, but may be seen in several other tumor cell types such as melanocytic lesions and selected histiocytic proliferations.[9] Indeed, the presence of S-100 immunoreactivity in spindle (neural) cells of our first case was initially thought to indicate cells of histiocytic origin.[9] Staining of spindle cells with macrophage markers in this case was misleading. The monoclonal antibody CD68 (KP1) reacts not only with fibrohistiocytic lesions, but also melanocytic lesions, neural tumors, lymphoma and some epithelial neoplasms.[10‐11] A high incidence of smooth-muscle tumors (leiomyomas and leiomyosarcomas) has been reported in association with HIV infection, including children with AIDS.[12‐14] HIV-associated leiomyosarcomas have been reported to occur in uncommon locations such as the lung, pericardium, pleura, spleen, adrenal gland, lymph node and orbit.[12] A potential role for EBV has been suggested in the tumorigenesis of HIV-associated leiomyosarcomas. Therefore, along with smooth muscle markers, stains to demonstrate EBV (LMP and/or EBER) infection within smooth muscle cells may be of diagnostic aid.

Patients infected with HIV are also at increased risk for fungal opportunistic infections. Several of these fungal infections may be dimorphic (i.e., exhibit fungal and yeast phases), such as pulmonary histoplasmosis caused by *Histoplasma capsulatum*. The identification of hyphae or yeast forms on cytological smears takes into account morphological criteria (e.g., size, shape, type of budding in the yeast forms) and additional staining characteristics.[15] Round to oval fungal forms are a common morphologic finding of *H.capsulatum* (size range 2–5 μm), *Cryptococcus neoformans* (size range 5–15 μm), and *Blastomyces dermatidis* (size range 8–20 μm). Several of these fungi may also demonstrate budding (e.g., narrow-based budding of *H.capsulatum* versus broad-based budding of *Blastomyces*), a thick capsule (e.g., *Cryptococcus neoformans*) or a thick refractile wall (e.g., *Blastomyces dermatidis*). In case 2, fragmented calcific debris that formed round bodies was initially mistaken for potential fungal forms. However, unlike fungi these calcified structures did not stain with mucicarmine or GMS stains. Intrinsic and extrinsic contaminants (e.g., airborne fungal spores and pollen)[16] and even crystals[17] may be mistaken for microorganisms, especially in the HIV positive host.

Pleural disease encountered in the HIV-infected patient may be due to infection (mycobacteria, bacterial pneumonia and more rarely other microorganisms like pneumocystosis), malignancy (lymphoma, KS and carcinoma), Castleman's disease, or may accompany systemic problems (for example, heart or renal failure).[18‐20] In the USA, the prevalence of HIV-related pleural effusion among hospitalized AIDS patients is 2-20%.[19] AIDS-related lymphomas involving pleural effusions may be primary (i.e., there is no extracavitary involvement or identifiable contiguous mass) or secondary (i.e., preceded by an extracavitary systemic lymphoma).[20] In case 3, our patient had a history of KS. Bloody or chylous pleural effusion occurs in approximately ^1^/_3_ to ^2^/_3_ of patients with KS[21‐22] and it was unclear in this particular case if KS contributed to his effusions. The presence of atypical cells in the pleural fluid of case 3 was very concerning for classic PEL. Other entities within the differential diagnosis were metastatic carcinoma, atypical/malignant mesothelial cells, and other neoplasms (e.g., melanoma and sarcoma). PEL is a high-grade non-Hodgkin lymphoma of B-cell origin that is strongly associated with HHV8 infection. Pre-existing KS is often reported in a large proportion of patients with PEL.[23] Cytologically, PEL cells are large atypical cells that range from immunoblast-like to anaplastic forms. Immunophenotypically these lymphoma cells are CD45, CD30 and LNA-1 (HHV8) positive, lack B-cell and T-cell antigens in the majority of cases, coexpress plasma cell markers (e.g., CD138), and are variably positive for EMA. In case 3, the atypical cells proved to be megakaryocytes, a component of this patient's extramedullary hematopoiesis. Extramedullary hematopoiesis is the formation and development of blood cells outside of the bone marrow. There have been several prior reports of patients presenting with pulmonary extramedullary hematopoiesis, most of which were due to secondary processes such as myeloproliferative disorders, hemolytic anemias, and hereditary spherocytosis.[24‐25] Although the driving force for extramedullary hematopoiesis in our patient was unclear, he did subsequently develop multiple myeloma.

In summary, we present three cases which illustrate how knowledge of a patient's HIV status was misleading in the evaluation of varying cytology specimens, with potential for misdiagnosis and/or multiple unnecessary procedures. In the setting of HIV infection, common entities unrelated to HIV infection must be maintained within the differential diagnosis. Contaminants and artifacts in these patients have the potential to masquerade and mimic significant infections. The use of ancillary studies including cytochemical stains and immunocytochemical studies are invaluable in such cases to establish an accurate diagnosis.

## COMPETING INTEREST STATEMENT BY ALL AUTHORS

No competing interest to declare by any of the authors.

## AUTHORSHIP STATEMENT BY ALL AUTHORS

Each author acknowledges that this final version was read and approved. All authors of this article declare that we qualify for authorship as defined by ICMJE http://www.icmje.org/#author. Each author has participated sufficiently in the work and take public responsibility for appropriate portions of the content of this article.

## ETHICS STATEMENT BY ALL AUTHORS

As this is case report without identifiers, our institution does not require approval from Institutional Review Board (IRB) (or its equivalent)

## EDITORIAL / PEER-REVIEW STATEMENT

To ensure integrity and highest quality of CytoJournal publications, the review process of this manuscript was conducted under a double blind model(authors are blinded for reviewers and reviewers are blinded for authors)through automatic online system..
